# Drug - related emergency department visits by elderly patients presenting with non-specific complaints

**DOI:** 10.1186/1757-7241-21-15

**Published:** 2013-03-05

**Authors:** Christian H Nickel, Juliane M Ruedinger, Anna S Messmer, Silke Maile, Arno Peng, Michael Bodmer, Reto W Kressig, Stephan Kraehenbuehl, Roland Bingisser

**Affiliations:** 1 Department of Emergency Medicine, University Hospital Basel, Basel, Switzerland; 2 Department of Pharmacology and Toxicology, University Hospital Basel, Basel, Switzerland; 3 Department of Acute Geriatrics, University Hospital Basel, Basel, Switzerland

## Abstract

**Background:**

Since drug-related emergency department (ED) visits are common among older adults, the objectives of our study were to identify the frequency of drug-related problems (DRPs) among patients presenting to the ED with non-specific complaints (NSC), such as generalized weakness and to evaluate responsible drug classes.

**Methods:**

Delayed type cross-sectional diagnostic study with a prospective 30 day follow-up in the ED of the University Hospital Basel, Switzerland. From May 2007 until April 2009, all non-trauma patients presenting to the ED with an Emergency Severity Index (ESI) of 2 or 3 were screened and included, if they presented with non-specific complaints. After having obtained complete 30-day follow-up, two outcome assessors reviewed all available information, judged whether the initial presentation was a DRP and compared their judgment with the initial ED diagnosis. Acute morbidity (“serious condition”) was allocated to individual cases according to predefined criteria.

**Results:**

The study population consisted of 633 patients with NSC. Median age was 81 years (IQR 72/87), and the mean Charlson comorbidity index was 2.5 (IQR 1/4). DRPs were identified in 77 of the 633 cases (12.2%). At the initial assessment, only 40% of the DRPs were correctly identified. 64 of the 77 identified DRPs (83%) fulfilled the criteria “serious condition”. Polypharmacy and certain drug classes (thiazides, antidepressants, benzodiazepines, anticonvulsants) were associated with DRPs.

**Conclusion:**

Elderly patients with non-specific complaints need to be screened systematically for drug-related problems.

**Trial Registration:**

ClinicalTrials.gov: NCT00920491

## Background

Drug-related ED visits are common. Up to 25% of ED consultations by elderly patients are due to drug-related problems (DRPs), depending on the definitions of DRP used [1-6]. Older patients are more frequently affected by DRPs than younger ones [1,2,7]. Contributing factors are physiologic changes associated with aging, which include impaired renal and hepatic function, as well as decreased total body water and lean body mass [8]. Additionally, older adults use more medications because of the co-existence of multiple comorbidities [9,10]. As a consequence, polypharmacy is highly prevalent in the older patient [11]. Furthermore, the presence of dementia or confusion may lead to patient errors due to complex medication regimens, resulting in DRPs [12,13].

Emergency Physician recognition of DRPs appears to be dependent on the mode of presentation [14]. Patients with DRPs can present to the ED with specific symptoms, e.g. rash, bleeding, arrhythmias, or hypoglycemia [15,16]. However, patients with DRPs may also present with non-specific complaints (NSC), such as generalized weakness which may make a DRP more difficult to be identified, considering the fact that ED physicians are failing to identify DRPs in up to 40% even in a general ED population [3].

Current research on DRPs in older patients is focused on falls and delirium [17,18], but up to 20% of elderly patients present to the ED with NSC, and the underlying cause of their symptoms is often not known [19,20]. Importantly, patients with NSC are at risk of adverse health outcomes [21-23]; probably because of their often older age, cognitive and functional impairment, multiple comorbidities, and sub-acute or atypical presentation of acute illness [19,24,25]. This patient group with NSC poses significant challenges to emergency physicians (EPs), as the differential diagnostic spectrum is so wide, ranging from lack of social support to life-threatening disease [26,27]. The need for research and training in this area has been addressed [28,29].

Currently it is not known how many patients with NSC suffer from DRPs. DRPs represent an important, potentially preventable and curable condition [30]. It can be speculated that DRPs in patients with NSC may be overlooked. Therefore, the objectives of our study were to identify the frequency of DRPs among patients presenting to the ED with NSC, to evaluate drugs and classes being associated with such DRPs and to assess the proportion of missed DRPs in the initial assessment. An additional aim was to determine the frequency of DRPs associated with acute morbidity.

## Methods

### Study design

This present study is a predefined part of the prospective Basel-non-specific complaints (BANC) study [22] with diagnostic assessment after a 30 day follow-up period by a panel of experts, representing a delayed type cross-sectional study [31]. The study protocol was approved by the local ethics committee (http://www.ekbb.ch, Reference Number EKBB 73/07) and it is registered with Clinical Trials (http://www.clinicaltrials.gov, NCT00920491). It is in compliance with the Helsinki Declaration.

### Study setting and population

The study was carried out in the ED of the University Hospital Basel, Switzerland. The hospital is an urban 700-bed tertiary care center with an ED census of over 41’000 patients per year. From May 24th 2007 until April 15th 2009, all non-trauma patients 18 years of age or older with an Emergency Severity Index (ESI) level of 2 or 3 [32] presenting to the ED were consecutively screened for inclusion. The ESI, a 5-level triage tool with proven reliability and validity for the German translation [33] was used in order to exclude all patients with life-threatening conditions (ESI 1), as well as patients with conditions in which a full work-up was not intended (“see-and-treat” pathway, ESI 4 and 5). All ED patients were screened twice for inclusion: First by a triage-nurse in order to include all ESI 2 and ESI 3 patients and secondly each resident on duty screened all ESI 2 and ESI 3 patients for their presenting symptoms using a one-page screening tool. Enrolment was then carried out by 3 previously trained study physicians.

### Inclusion criteria

Patients were included if they presented with NSC. NSCs are defined as the entity of complaints which are not part of the set of specific complaints or signs or where an initial working diagnosis cannot be definitively established [22]. For such complaints, no evidence-based management protocols (EPs) exist. Typical examples of NSC are “generalized weakness”, “general deterioration” or “lack of community support” [34,35].

### Exclusion criteria

Patients with specific complaints (e.g. syncope, chest pain) were not included. Hemodynamically unstable patients or with vital parameters significantly out of the normal range (systolic blood pressure <80 or >180 mmHg, respiration rate >20/min, tympanal body temperature >38.5°C, SpO_2_ < 92%) were also excluded. Patients with abnormal vital signs can be considered as “specific”, since work-up is generally straight-forward, for example for shock, even when the symptoms are non-specific. In addition, patients with known terminal conditions as well as patients referred from other hospitals were not eligible for inclusion.

### Measurements

Demographic baseline data, ESI, all complaints (using a structured interview form), vital signs (pulse, blood pressure, respiratory rate, and oxygen saturation), Glasgow Coma Scale, medical history, physical examination, and electrocardiography reading were obtained on arrival and registered on the patient’s case report form. Additionally, the body mass index (BMI) was calculated. For quantification of comorbidities the Charlson Comorbidity Index was used [36]. This tool has been validated for population-based studies to estimate the risk of death. Lab testing was done in all patients and, in the vast majority, chest X-ray and urinalysis was performed. Treatment was initiated at the discretion of the ED physician in charge.

### Assessment of medication

We assessed all drugs reported by the patient during a bedside interview, or reported by proxies or by the family physician, or found in our electronic patient records. Drugs were grouped into predefined pharmacological classes (Table 1). Additionally, we analyzed the drugs recorded according to the Beers criteria, a list of potentially inappropriate medications in older adults which aims to minimize drug-related problems [37]. The Beers criteria are regularly updated [38] and based on expert consensus. They are developed through literature review and a questionnaire. They have been evaluated by recognized experts in geriatric care, clinical pharmacology and psychopharmacology.

**Table 1:** Prescribed drugs at presentation to the ED

**Drug class**	**Number of patients**	**Percent % (total n = 633)**
Diuretics (all types)	294	46.4
Non-thiazide diuretics	225	41.4
Thiazide diuretics	136	21.5
ACE inhibitors/AR blockers	231	36.5
Beta-blockers	211	33.3
Other cardiovascular drugs^†^	129	20.4
Platelet aggregation inhibitors	236	37.3
Vitamin K- antagonists	90	14.2
Systemic steroids	47	7.4
NSAID	98	15.5
Opioids	69	10.9
All psychotropic drugs	298	47.1
Benzodiazepines	121	19.1
Neuroleptics	91	14.4
Antidepressants	160	25.3
Other^‡^	86	13.6
Anticonvulsants	61	9.6
Anti-Parkinson drugs	48	7.6

^†^calcium channel blockers, nitrates, digoxine, amiodarone.

^‡^other hypnotica such as barbiturates, zopiclone and zolpidem and others, such as St. Johns Worth.

### Patient follow up and endpoint ascertainment

Written 30-day follow-up data was obtained from hospital discharge reports or the patients’ primary care physicians (questionnaire). Outcome assessors reviewed all available medical records and data of the study patients from the time of ED presentation to 30-day follow-up. Six ED physicians one of whom is a clinical pharmacologist, all certified in internal medicine and experienced in emergency medicine (outcome assessors) were available for outcome ascertainment. In each session, two outcome assessors, reviewed all discharge records and established a final “gold standard” diagnosis according to the 10th International Classification of Diseases and Related Health Problems (ICD-10). Furthermore, the diagnoses were evaluated to be drug-related or not using Micromedex [39]. For each medication entered, a detailed list of possible DRPs was produced using Micromedex. Where needed, a board-certified clinical pharmacologist was consulted. In the 22 cases of disagreement between outcome assessors, cases were reviewed and adjudicated in conjunction with the BANC expert panel, consisting of two physicians certified in internal medicine with at least 10 years of clinical experience as previously described [22].

In a second step, the expert panel classified the DRP according to the Pharmaceutical Care Network Europe (PCNE) Classification (see below) [40].

The DRPs identified as described above were compared with the ED diagnoses given on the day of the visit. By comparison, the proportion of missed DRPs could be identified.

### Definition and classification of drug-related problems (DRPs)

According to the PCNE Classification V 5.01, DRPs are defined as “events or circumstances involving drug therapy actually or potentially interfering with desired health outcomes” [40]. In this classification, DRPs can be assigned to six main categories, so-called “primary domains” P1-P6: (P1) adverse drug reaction (allergic, non-allergic or toxic side-effects), (P2) drug choice problem (patient gets or is going to get a wrong (or no drug) drug for his/her disease and/or condition), (P3) dosing problem (patient gets more or less than the amount of drug he/she requires), (P4) drug use problem (wrong or no drug taken/administered), (P5) drug-drug interactions, and (P6) others. We chose the PCNE classification, since it has been shown to contain all required aspects to describe and classify DRPs for research and practice purpose [41]. Furthermore, it has been evaluated in our institution and is considered to be a practical tool in the hospital setting [42]. Polypharmacy was defined as the prescription of 6 or more drugs at presentation [43].

### Serious condition

A “serious condition” was attributed to individual cases using predefined criteria that are covered in a comprehensive list [22]. Briefly, a “serious condition” was defined as a potentially life-threatening condition, or any condition requiring an early intervention (e.g. central nervous dysfunction due to hyponatremia) to prevent health status deterioration leading to potential morbidity or death within 30 days of the initial ED presentation.

### Data analysis

In case of categorical variables, crosstables were calculated. In case of ordinal or metric variables, mean, median, standard deviation, minimum, maximum were calculated. To predict the influence of the number of concomitantly prescribed drugs and of certain drug classes (e.g. diuretics, psychotropic drugs) on the outcomes DRP or DRP with “serious condition”, a logistic regression model was chosen. Adjustment for age, gender and comorbidities was performed by adding these cofactors to the regression model. Results are presented as two-sided p-values and odds ratios (ORs) with their corresponding 95% confidence intervals. In the case of continuous or ordinal predictors, the OR is expressed as the ratio of the odds increasing the predictor from the 1rd to the 3st quartile (25 and 75 percentile, respectively), representing a typical above average to a typical below average value. A p-value < 0.05 was considered significant. This study is exploratory; therefore p-values were not adjusted for multiple comparisons. All analyses were done using R v 2.8.0 (A Language and Environment for Statistical Computing).

## Results

**Figure 1:**
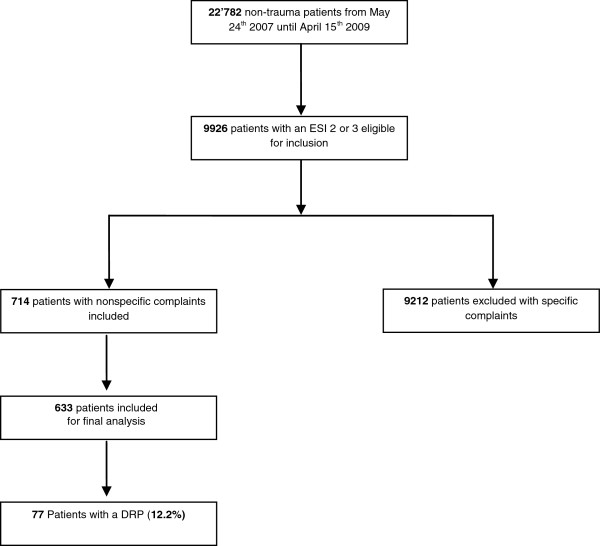
Study outline.

From May 24th 2007 until April 15th 2009, 22’782 non-trauma patients presented to the ED. 9926 patients were triaged as ESI level 2 or ESI 3 and therefore screened for inclusion into the current study. Of these, 714 patients (7.2%) presented with NSC and were consecutively enrolled in our study (see Figure 1). During post-hoc case reviews, the BANC expert panel recalled the inclusion of 81 patients due to the presence of exclusion criteria, mostly due to the presence of specific complaints. The final study population consisted of 633 patients with NSC. No patient was lost to follow-up. Baseline characteristics of the study population are presented in Table 2. Median age was 81 years (IQR 72/87), 62.6% of subjects were female, median BMI was 23.4 kg/m^2^ (IQR 20.4/26.4). Mean Charlson comorbidity index was 2.5 (IQR 1/4). 581 patients (91.7%) took 1 or more medications (prescribed, or over the counter). The number of medications taken ranged from 0 to 17 with a median number of 5 drugs (IQR 3/8). The detailed analyses of the drug categories are presented in Table 1.

**Table 2:** Baseline characteristics of the study population

**Characteristics**	**Total population (N = 633)**
Age, median (IQR)	81 (72/87)
Female (%)	396 (62.6)
BMI, median (IQR)	23.4 (20.4/26.4)
Living situation:	
- independent (%)	145 (22.9)
- with family help (%)	208 (32.9)
- with home care (%)	214 (33.8)
- nursing home (%)	66 (10.4)
Charlson Comorbidity Index, median (IQR)	2 (1/4)
Number of concomitant drugs, median (IQR)	5 (3/8)
Patients with serious condition, number (%)	387 (61.1)
Patients with DRP, number (%)	77 (12.2)
Patients with DRP as serious condition, number (%)	64 (83.1)

*IQR* = Inter-Quartile Range.

*BMI* = Body Mass Index.

*MDRD* = Modification of Diet in Renal Disease, equation to predict glomerular filtration rate from serum creatinine [44].

*DRP* = drug-related problems, as defined in the PCNE-Classification V5.1.

A total number of 77 patients with DRPs were identified, attributing to 12.2% of the total study population (633 patients). A “Serious Condition” as defined above was detected in 64 of the 77 identified DRPs (83%). Of the 633 patients with NSC screened for DRPs, 387 suffered from a “serious condition” (61%). The comparison of the 77 cases with a DRP with the initial ED diagnosis given on the day of the visit revealed that 48 cases with a DRP (60%) were initially not identified. Most commonly missed drug-related diagnoses were electrolyte disorders (hyponatremia and others, 20 cases), hypovolemia (7 cases) and intoxications (5 cases).

**Figure 2:**
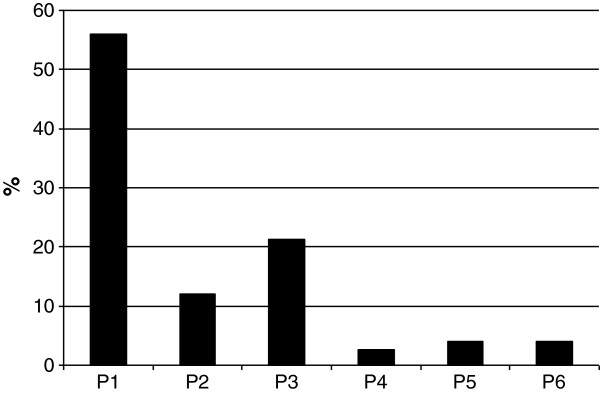
**PCNE Classification and percentage of all our identified drug-related problems (DRP), N = 77.** It has six primary domains for problems: P1 = adverse drug reactions, P2 = Drug choice problem, P3 = Dosing problem, P4 = Drug use problem, P5 = Interactions, P6 = other.

42 DRPs (56% of all DRPs) were classified under the P1 domain “adverse drug reactions (ADR)”. Nine DRPs (12% were classified as P2 (drug choice problem), 16 (21%) as P3 (dosing problems), 2 (3%) as P4 (drug use problems), 3 (4%) as P5 (drug interactions) and 3 (4%) as P6 (other) (see Figure 2).

### Drugs associated with DRP

In our population, each additional drug accounted for an increase of 10% in the probability of suffering from a DRP. This association was observed for treatment with 3 to 8 drugs.

In patients presenting with NSC, the use of psychotropic drugs was significantly associated with DRPs (adjusted OR 2.32, 95% CI 1.39 - 3.88, p = 0.001). This association became stronger with an increasing number of the psychotropic drugs used (adjusted OR for one psychotropic drug.1.89, 95% CI 1.06 - 3.37, p < 0.05 and for 2 psychotropic drugs 2.95, 95% CI 1.51 - 5.76, p < 0.01). Both the intake of benzodiazepines and antidepressants were associated with DRPs (p < 0.001) in patients with NSC with an adjusted OR of 2.5 (95% CIs 1.47 - 4.28) and 2.41 (1.46 - 3.98), respectively. Furthermore the use of anticonvulsants (adjusted OR 3.06, 95% CI 1.55 - 6.02, p < 0.05) and of diuretics (adjusted OR 1.8, 95% CI 1.06 - 3.04, p < 0.05) was associated with an increased risk for DRPs. Further analysis of the risk associated with diuretics showed that the use of non-thiazide diuretics did not increase the risk for DRPs significantly (adjusted OR 1.54, 95% CI 0.92 - 2.59, p = 0.1), but that the risk for DRPs was increased with the use of thiazides (adjusted OR 2.09; 95% CI 1.21-3.62, p = 0.009) (see Table 3). In 7 cases, more than one drug was found to be associated with the respective DRP. Other drug-classes (e.g. NSAIDs, betablockers, neuroleptics, opioids) were not significantly associated with DRPs in our study population with NSC (data not shown).

**Table 3:** Observed DRPs

**Involved organ system**	**Involved drugs**^**†**^
**Electrolytes**	
Hyponatremia	Thiazide diuretics (14), Non-thiazide diuretics (2), Anticonvulsants (2), Antidepressants^±^ (2), ACE/ARB blockers (1)
Hypokalemia	Non-thiazide diuretics (3), Thiazide diuretics (1)
Hyperkalemia	ACE/ARB blockers (1)
**Cardiovascular**^‡^	
Bradycardia	Beta blockers (1)
Hypotension	Beta blockers (1)
Acute heart failure	NSAID (1)
Hypovolemia/orthostatic dysregulation	Thiazide diuretics (7), Non-thiazide diuretics (4),
**Kidney**	
Hypovolemia, prerenal azotemia	Non-thiazide diuretics (3), thiazide diuretics (1)
Acute kidney injury	ACE/ARB blockers (1), NSAID (3)
**Coagulation system**	
Intracranial hemorrhage	Vitamin-K antagonists (1)
**Gastrointestinal**	
Nausea	Opioids (1), Antibiotics (1), Interferon γ (1)
Gastric ulcer/Gastritis	NSAID (2)
**Central nervous system**	
Aggravation of Parkinson’s	Underdosing Anti-Parkinson drugs (1)
Status epilepticus	Malcompliance anticonvulsants (2)
**Hematology**	
Neutropenic complication	Chemotherapy (1)
Anemia	Underdosing of Darbepoetin alpha (1)
**Endocrine**	
Diabetes mellitus	Systemic steroids (1)
Addison	systemic steroids (1)
**Other**	
Overdosing	Opioids (7), Benzodiazepines (5), Anticonvulsants (3), Neuroleptics (1)
Falls	Neuroleptic (1)
Tumor lysis syndrome	Chemotherapy (1)
Abusus	Laxatives (1)

^†^In 7 cases, the DRP was caused by more than one drug class. In 2 cases, one drug caused more than one DRP.

^±^SSRI, SNRI, Tricyclic antidepressants.

^‡^Vital signs within normal range at presentation.

### Corresponding final diagnosis

The most frequent underlying causes of DRPs with NSC were hyponatremia (27%) and drug-overdose (20%). Hyponatremia was most commonly due to thiazides as well as due to the syndrome of inappropriate secretion of antidiuretic hormone (SIADH) associated with antidepressants or anticonvulsants. Drug-overdose was mostly due to prescribed benzodiazepines or opiates. A more detailed list of the corresponding final diagnosis is shown in Table 3.

## Discussion

To our knowledge, this is the first study assessing DRPs in elderly ED patients with non-specific complaints. Our results show a prevalence of 12.2% for DRPs in this patient group. Thus, DRPs rank among the top five causes for non-specific ED presentations, with the vast majority (83%) causing acute morbidity, classified as a “serious condition”. This illustrates the importance of early detection of DRPs in the ED. In our study, 60% of DRPs in patients visiting the ED with NSC were initially not diagnosed as medication-related, and were also not considered in the differential diagnosis.

The risk for a DRP increased with the number of prescribed medications and with treatment with certain drug classes, in particular antidepressants, benzodiazepines and anticonvulsants. Intake of thiazides, benzodiazepines, antidepressants and anticonvulsants was associated with a significantly increased risk for DRPs. The corresponding final diagnosis was most commonly hyponatremia or medication-overdose.

Non-specific complaints are a common mode of presentation in the ED and have previously been described using terms, such as “general deterioration”, “loss of energy”, “weakness” or “home care impossible” [19,20,34,45]. Elderly patients with comorbidities, who are not institutionalized, belong to the high-risk population developing DRPs [4,12,46]. This risk group is highly represented in our study population. The vast majority of our patients with NSCs were not institutionalized (89.6%) and had a relatively high burden of comorbidity compared to similar populations described [47,48].

It has been previously shown that for an elderly ED population the most common drugs causing DRPs are diuretics, oral anticoagulants, NSAIDs, antiarrhythmics, antiplatelet agents and psychotropic drugs [49]. In another study on older patients, Warfarin, Insulin, oral antiplatelet agents and oral hypoglycemic agents were implicated alone or in combination in 67% of emergency hospitalizations for adverse drug events [50]. However, drug classes such as anticoagulants, NSAIDs, antiarrhythmics, and antiplatelet drugs tend to cause specific symptoms (e.g. bleeding, syncope) or signs (e.g. bradycardia, hypotension) in contrast to diuretics. This may explain the under-representation of DRPs induced by these substances in our study population whose chief complaints were merely non-specific.

In accordance with the Beers criteria, we observed several cases of DRPs due to SSRIs, benzodiazepines and anticonvulsants [37]. However we also detected DRPs associated with drugs which are neither listed in the Beers criteria of 2003 nor 2012, e.g. thiazide-diuretics [38]. This discrepancy which was also observed in other studies [51-53], could be due to a different prescribing pattern or differing opinions about inappropriateness between the US and Europe [54,55]. Another publication compared seven explicit criteria of drug inappropriateness in elderly patients from different countries, including the Beers criteria [56]. The only drugs considered inappropriate by all seven criteria were long-acting benzodiazepines and tricyclic antidepressants [56]. Diuretics were listed by four of the seven criteria, mainly as potentially inappropriate in combination with other drugs (e.g. NSAIDs, digoxin) or in patients with a history of gout [57-60].

Importantly, one study revealed that up to 40% of drug-related ED visits are not correctly diagnosed by Emergency Physicians (EP) in a general ED population [3]. Obviously, DRPs can be difficult to diagnose for EPs, especially when elderly patients present with non-specific complaints. In this subgroup of patients, the proportion of initially missed DRPs was even higher in our study. A potential reason for this might be that EPs are better at identifying DRPs which relate to the patients’ chief complaints as compared to DRPs with symptoms unrelated to their chief complaints [7]. However, NSCs were not specifically addressed in that study. The high proportion of DRPs that were not identified in our study strongly supports the hypothesis that non-specific manifestations of DRPs are more likely to be missed on admission than DRPs associated with specific symptoms.

## Limitations

Potential limitations of our study include that this study was conducted at a single urban tertiary care centre in Switzerland. The lack of an external validation sample limits the generalizability of the results. Furthermore, age and gender could not be disguised from the outcome assessors, leaving the possibility of some degree of incorporation bias [31]. Also, the definition of non-specific disease presentation might not be generalizable to other settings. Furthermore, we may have missed some cases of patients with NSC during enrolment (no informed consent given; non-inclusion during ED crowding). However, since the data were obtained in a large sample of consecutive ED patients, our NSC cohort appears stable when comparing the different parts of the BANC study in terms of demographic data, rates of acute morbidity, and mortality rate [22,23].

The results of this study are well in accordance with previous investigations demonstrating that a relevant number of drug-related ED visits are undetected [3,46]. Furthermore, the identification of DRPs in patients with non-specific complaints seems to be even more difficult than for a general ED population. However, this study here was not designed to investigate this question, as the proportion of missed DRPs in specific complaints was not recorded. An underestimation of DRPs by the expert panel is possible, especially as the clinical pharmacologist was not involved in outcome assessment of all 633 cases. However, other studies have demonstrated similar frequencies of DRPs in ED patients [3,46,61]. Additionally, we did not assess for the individual preventability of DRPs in this exploratory study.

The risk of falls is known to be significantly associated with psychotropic medication, especially for benzodiazepines and other hypnotics, as well as for antiepileptics [62]. However, trauma patients with injurious falls were not included in our study.

## Conclusion

The ED is an environment where medication regimens of elderly patients need to be screened systematically for drug-related problems due to both the frequency and the possible severity of the condition. Patients with non-specific complaints are known to be at high risk for complications – our findings add DRPs to the long list of possible complications and missed conditions in this population. It is important to realize that the number of medications and treatment with specific drugs or drug classes such as thiazides, benzodiazepines, antidepressants, and anticonvulsants are significantly associated with DRPs, resulting in ED visits with non-specific complaints. Better knowledge of these relationships may help to decrease the frequency of missed DRPs in this patient group.

## Competing interests

The authors declare that they have no competing interests.

## Authors’ contributions

CHN, MJR, SM, RB study concept and design. CHN, MJR, SM, ASM acquisition of subjects and data. CHN, MJR, SM, AP, MB, RWK, SK, RB analysis and interpretation of data, and preparation of manuscript. (See section on “Authorship and Duplicate Publication”). All authors read and approved the final manuscript.
